# Improved Image Quality of Virtual Monochromatic Images with Deep Learning Image Reconstruction Algorithm on Dual-Energy CT in Patients with Pancreatic Ductal Adenocarcinoma

**DOI:** 10.1007/s10278-025-01514-6

**Published:** 2025-04-30

**Authors:** Keitaro Sofue, Eisuke Ueshima, Yoshiko Ueno, Takeru Yamaguchi, Masatoshi Hori, Takamichi Murakami

**Affiliations:** 1 Department of Radiology, Kobe University Graduate School of Medicine, 7-5-2, Kusunoki-Cho, Chuo-Ku, Kobe, 650-0017 Japan; 2 Department of Artificial Intelligence in Diagnostic Radiology, Osaka University Graduate School of Medicine, Osaka, Japan

**Keywords:** Dual-energy CT, Virtual monochromatic images, Deep learning image reconstruction, Pancreatic ductal adenocarcinoma

## Abstract

This study aimed to evaluate the image quality of virtual monochromatic images (VMIs) reconstructed with deep learning image reconstruction (DLIR) using dual-energy CT (DECT) to diagnose pancreatic ductal adenocarcinoma (PDAC). Fifty patients with histologically confirmed PDAC who underwent multiphasic contrast-enhanced DECT between 2019 and 2022 were retrospectively analyzed. VMIs at 40–100 keV were reconstructed using hybrid iterative reconstruction (ASiR-V 30% and ASiR-V 50%) and DLIR (TFI-M) algorithms. Quantitative analyses included contrast-to-noise ratios (CNR) of the major abdominal vessels, liver, pancreas, and the PDAC. Qualitative image quality assessments included image noise, soft-tissue sharpness, vessel contrast, and PDAC conspicuity. Noise power spectrum (NPS) analysis was performed to examine the variance and spatial frequency characteristics of image noise using a phantom. TFI-M significantly improved image quality compared to ASiR-V 30% and ASiR-V 50%, especially at lower keV levels. VMIs with TFI-M showed reduced image noise and higher pancreas-to-tumor CNR at 40 keV. Qualitative evaluations confirmed DLIR's superiority in noise reduction, tissue sharpness, and vessel conspicuity, with substantial interobserver agreement (κ = 0.61–0.78). NPS analysis demonstrated effective noise reduction across spatial frequencies. DLIR significantly improved the image quality of VMIs on DECT by reducing image noise and increasing CNR, particularly at lower keV levels. These improvements may improve PDAC detection and assessment, making it a valuable tool for pancreatic cancer imaging.

## Introduction

According to the National Comprehensive Cancer Network (NCCN) guidelines, multiphasic contrast-enhanced CT (CECT) is the recommended imaging tool for evaluating local resectability and distant metastasis in pancreatic ductal adenocarcinoma (PDAC) patients [1, 2]. Pancreatic phase (PP) images aid in tumor detection and assessment of arterial involvement, while portal venous phase (PVP) images facilitate the evaluation of peripancreatic veins and liver metastases [3, 4]. However, conventional 120-kilovolt peak (kVp) cannot identify PDAC in some cases due to the reduced contrast between tumor and adjacent pancreatic parenchyma [5, 6]. To improve contrast for PDAC diagnosis, various approaches, including higher iodine concentration, low tube-voltage CT, delayed phase imaging, and dual-energy CT, have been investigated [7–10].

Virtual monochromatic images (VMIs) from dual-energy CT (DECT) can generate CT images at various energy levels and have demonstrated added value in abdominal evaluation [11–13]. Low-kilo-electron volt (keV) VMI increases contrast enhancement of tissues and vasculature, improving PDAC detection and assessment [14, 15], because iodine attenuation increases as the energy approaches the iodine K-edge of 33.2 keV [11–13]. However, a disadvantage of VMI is increased image noise, especially in thin-slice low-keV VMI, which limits its diagnostic utility [14, 15].

Deep learning image reconstruction (DLIR) algorithms (TrueFidelity, GE Healthcare) incorporating deep convolutional neural networks into the image reconstruction process were introduced. In this algorithm, ground truth training data were obtained from high dose filtered back projection (FBP) images of phantoms in the laboratory and clinical participants [16]. Compared to FBP and iterative reconstruction algorithms, DLIR can reduce image noise and thus improve image quality without altering texture [17–19]. In addition, DLIR has been applied to both DECT and single-energy CT imaging. We hypothesized that VMI with DLIR algorithms could further reduce image noise and enhance image quality in pancreatic DECT compared to other reconstruction algorithms.

This study aimed to evaluate the image quality of VMIs reconstructed with DLIR using DECT to diagnose PDAC.

## Materials and Methods

### Study Population

This study was a retrospective analysis of a prospectively collected cohort, approved by the Institutional Review Board, with a waiver of written informed consent. Fifty-three consecutive patients with histologically confirmed PDAC underwent multiphasic contrast-enhanced CT of the pancreas on single-source DECT for initial diagnosis and staging between January 2019 and January 2022. Three patients were excluded because the PDAC was located at the end of the pancreatic head, precluding the measurement of pancreatic parenchymal CT values downstream of the tumor. The final study population included 50 patients (mean age, 68.0 years; range, 44‒88 years) with 36 men (mean age, 67.4 years; range, 44‒86 years) and 14 women (mean age, 69.4 years; range, 55‒88 years). None of the participants received chemotherapy or radiotherapy before the CT scans. Other information, including body weight, body mass index, and tumor characteristics, was obtained from the electronic medical records.

### DECT Acquisition and Image Reconstruction

All CT images were acquired on a DECT system (Revolution CT, GE Healthcare) in dual-energy mode with fast kilovoltage switching between 80 and 140 kVp in adjacent views during a single rotation. Acquisition settings were as follows: noise index, 9 Hounsfield units (HU) at a 5 mm slice collimation; tube current, variable (GSI Assist; GE Healthcare); primary reconstruction, 5 mm at 70 keV with 30% adaptive statistical iterative reconstruction Veo (ASiR-V; GE Healthcare); detector configuration, 128 × 0.625 mm; beam collimation, 80 mm; acquisition matrix, 512 × 512; gantry rotation speed, 0.5 s; and pitch factor, 0.508. An iodinated contrast material (600 mg I/kg) was injected into the antecubital vein for a fixed duration of 30 s, followed by flushing with 30 mL of saline using a mechanical power injector. All scans included PP images acquired with a delay of 20 s after a trigger threshold (80 HU) in the abdominal aorta at the level of the first lumbar vertebral body using a bolus tracking program (SmartPrep, GE Healthcare), followed by PVP images obtained 30 s after the end of the PP.

Raw data from PP and PVP images were transferred to a post-processing workstation (AW; GE Healthcare), and all data were reconstructed in the axial plane with a 1.25-mm thickness and 1.25-mm interval using a standard soft-tissue kernel. Using projection-based material decomposition software, VMI at 40‒100 keV (VMI_40‒100_, 10 keV increments) was generated in each patient with the following three reconstruction algorithms: hybrid-iterative reconstruction (ASiR-V 30%, ASiR-V 50%), and DLIR (TrueFidelity™; GE Healthcare) at medium strength level (TFI-M).

Radiation doses were estimated from PP and PVP images. The volume CT dose index (CTDI_vol_) and the dose-length product (DLP) presented by the CT equipment were documented.

### Quantitative Image Analysis

**Fig. 1:**
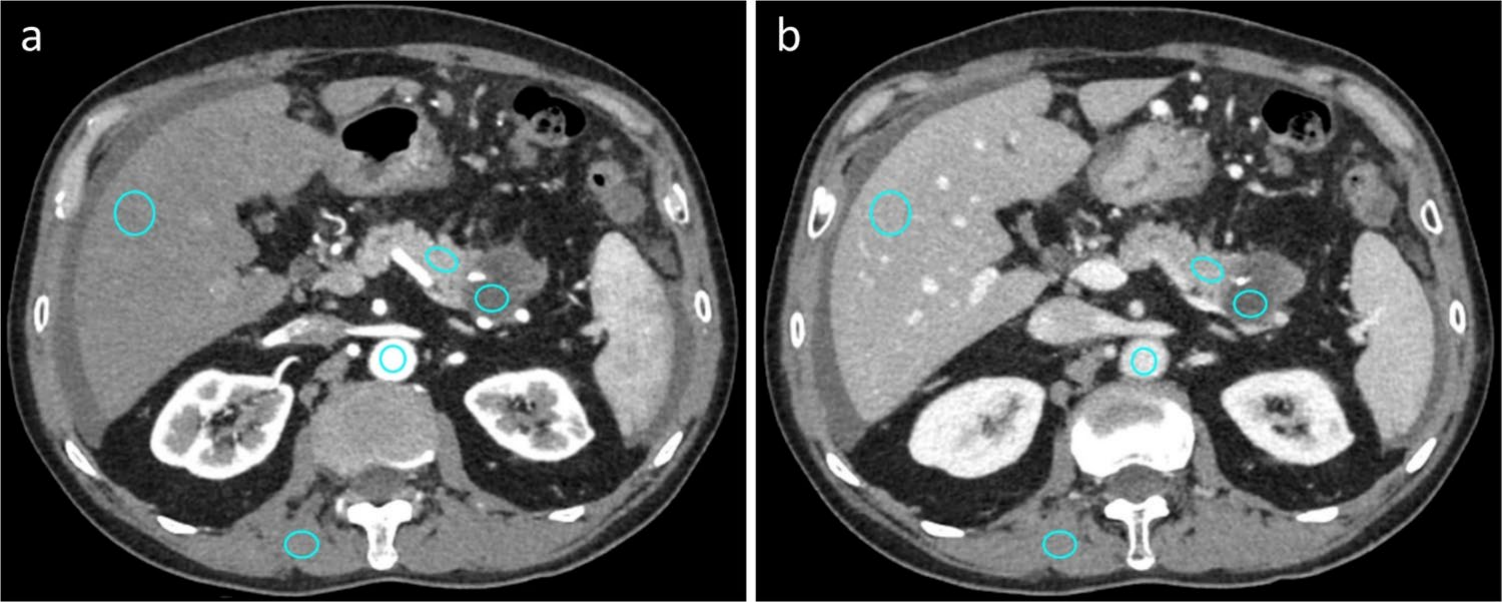
Virtual monochromatic images at 70 kiloelectron-volt (keV) reconstructed using a deep-learning image reconstruction algorithm in the **(a)** pancreatic and **(b)** portal venous phase images. To measure CT values, regions of interest were manually drawn on the paraspinal muscle, abdominal aorta, portal vein, liver, pancreas, and pancreatic ductal adenocarcinoma

Quantitative image analysis was performed in consensus by one radiologic technologist and one board-certified abdominal radiologist (K.S., with 17 years of experience in abdominal imaging). For the CT attenuation measurements, regions of interest (ROIs) were placed within the aorta, portal vein, liver, pancreas, pancreatic ductal adenocarcinoma (PDAC), and paraspinal muscles (Fig. 1). Aortic CT attenuation was measured only on PP images at the level of the first lumbar vertebral body to avoid calcification and soft plaques. The ROI within the portal vein was placed only on PVP images at the confluence levels of the right and left portal veins. CT attenuation of the liver was recorded as the mean measurement of the right anterior and posterior segments of the liver, avoiding focal lesions, large vessels, and areas of focal hepatic parenchymal changes. CT attenuation of the pancreas was measured at the segment downstream of the PDAC while carefully avoiding the visible pancreatic duct and vessels [20]. For PDAC measurement, a circular ROI was placed as large as possible on the images showing the tumor's maximum diameter. For the paraspinal muscle, the ROI was maintained at the level of the lumbar vertebral body to avoid visible fatty infiltration, and the standard deviation (SD) and CT attenuation were recorded as image noise. All quantitative measurements were performed twice at each location to confirm consistency, and the average values were used for the analysis.

For the PP and PVP images in VMI_70_ for each reconstruction algorithm, the contrast-to-noise ratio (CNR) of the aorta, portal vein, liver, and pancreas were calculated using the following equations:

$${\mathrm{CNR}}_{\mathrm{object}}=\left({\mathrm{HU}}_{\mathrm{object}}-{\mathrm{HU}}_{\mathrm{muscle}}\right)/\text{image noise}$$

The pancreas-to-tumor CNR was calculated from the PP and PVP images in VMI_40‒100_ using the following equation:

$${\mathrm{CNR}}_{\mathrm{PDAC}}=\left({\mathrm{HU}}_{\mathrm{pancreas}}-{\mathrm{HU}}_{\mathrm{PDAC}}\right)/\text{image noise}$$

where HU_object_, HU_muscle_, HU_pancreas_, and HU_PDAC_ represent the CT attenuation of the object, paraspinal muscle, pancreas, and PDAC, respectively, and the image noise refers to the SD of the paraspinal muscle.

Additionally, two board-certified radiologists (K.S. and E.U., with 18 and 14 years of experience in abdominal imaging, respectively) independently measured maximum diameter of the PDAC in VMI_40‒100_ on PP images in each reconstruction algorithm to evaluate inter-reader variability.

### Qualitative Image Analysis

Two board-certified radiologists (K.S. and E.U.) independently performed qualitative image analysis. Each reader was aware that the patients had pathologically proven PDAC but was blinded to other patient information and acquisition parameters, including reconstruction algorithms. For image analysis, each reader reviewed VMI_70_ images and the VMI_40_ to evaluate the subjective image quality, including image noise, soft tissue sharpness, vessel contrast, conspicuity of PDAC, and overall image quality by grading on a 5-point Likert scale: image noise (1 = undiagnostic; 2 = suboptimal; 3 = moderate; 4 = mild; and 5 = absent), soft tissue sharpness (1 = unclear; 2 = suboptimal; 3 = acceptable; 4 = good; and 5 = excellent), vessel contrast (1 = undiagnostic [poor boundary delineation]; 2 = suboptimal [visible but insufficient contrast]; 3 = acceptable [visible and sufficient for diagnosis]; 4 = good [well delineated with sufficient contrast]; and 5 = excellent [sharp vessel visualization with excellent contrast]), conspicuity of PDAC (1 = undiagnostic; 2 = suboptimal; 3 = acceptable; 4 = good; 5 = excellent), and overall image quality (1 = unacceptable diagnostic image quality; 2 = subdiagnostic; 3 = average; 4 = above average; 5 = excellent). The reason why VMI_40_ was used for this analysis is that a previous study reported VMI_40_ as an optimal energy level for the assessment of tumor-to-pancreas CNR [20], and our quantitative image analysis also showed that the highest pancreas-to-tumor CNR was achieved on VMI_40_ with any reconstruction algorithms. Images were presented in random order with a preset soft-tissue window setting (350 HU width and 40 HU level), and the readers could adjust the window setting at their discretion. The average scores of the two readers were statistically analyzed.

Diagnostic performance in determining surgical resectability of PDAC was assessed in VMI_40_ and VMI_70_ reconstructed with TFI-M in patients who underwent surgical resection. Two radiologists classified tumor involvement as resectable, borderline resectable, or unresectable according to the National Comprehensive Cancer Network criteria [1]. In addition, possibility of R0 resection was also assessed using a 5-point scale (1 = definitely possible R0 resection; 2 = probably possible R0 resection; 3 = indeterminate for R0 resection; 4 = probably impossible for R0 resection; 5 = definitely impossible for R0 resection. Resectable tumors were assigned as a score of 1, and unresectable tumors were assigned a score of 5 based on the NCCN criteria. In case of borderline resectable tumors, a score of 2–4 was assigned based on the degree of tumor vessel contact [4].

### Phantom Experiment

In addition to patient examinations, a phantom experiment was performed to examine the variance and spatial frequency characteristics of image noise. The noise power spectrum (NPS) was measured using a vendor-specific, commercially available water quality assurance phantom (GE Healthcare), 24 cm in diameter, designed to simulate the attenuation of water. The phantom was scanned with a CT scanner using the same acquisition parameters with CTDI_vol_ levels of 9 and 15 mGy. The VMI_70_ was reconstructed in the axial plane with 1.25-mm thickness and 1.25-mm reconstruction interval with ASiR-V 30%, ASiR-V 50%, and TFI-M reconstruction algorithms.

The NPS was obtained using the radial frequency method with a square ROI of 256 × 256 pixels placed at the center of each image. Two-dimensional trend removal and averaging over the entire circumference direction using a two-dimensional fast Fourier transform were applied [21–23]. Measurements were performed for each radiation dose level and reconstruction algorithm, utilizing five scans in uniform sections of the phantom, and the mean values were applied for the assessment.

### Statistical Analysis

Continuous variables were expressed as mean ± standard deviation and categorical variables were summarized as counts and frequencies. The Shapiro–Wilk test was used to confirm the normality of the data distribution.

For quantitative analyses, repeated-measures analysis of variance test with Bonferroni correction was used to evaluate differences in the HU values, image noise, CNR of each object at VMI_40_, and the pancreas-to-tumor CNR at VMI_40‒100_ among the ASiR-V 30%, ASiR-V 50%, and DLIR-M algorithms. Inter-reader agreements in measured values were assessed by using intraclass correlation coefficient (ICC). The ICC values were interpreted as poor (0–0.50), moderate (0.51–0.75), good (0.75–0.90), or excellent (0.90–1.00). For qualitative analyses, the Kruskal–Wallis test was used to compare the grading scales for soft tissue sharpness, vessel contrast, conspicuity of PDAC, and overall image quality among the ASiR-V 30%, ASiR-V 50%, and DLIR-M algorithms. The area under the receiver operating characteristic curve (AUC) was measured to assess the diagnostic performance of VMI40 and VMI70 with TFI-M in determining surgical resectability. Sensitivity and specificity were calculated using scores 1–3 of R0 resection probability to indicate the R0 resection. Interobserver variability for qualitative analysis was assessed using the linear-weighted ĸ statistic for each assessment. The κ values of 0.01–0.20 were considered to indicate slight agreement, 0.21–0.40 fair agreement, 0.41–0.60 moderate agreement, 0.61–0.80 substantial agreement, and 0.81–1.00 almost perfect agreement.

Statistical analyses were performed using the R software (version 3.4.1; R Foundation for Statistical Computing, Vienna, Austria). A two-sided *P* < 0.05 was considered statistically significant.

## Results

### Study Population

The patient characteristics are presented in Table 1. The patients'mean body weight was 60.5 ± 10.8 kg, and the mean body mass index was 22.5 ± 3.2 kg/m^2^. PDAC was diagnosed using fine-needle aspiration (n = 21) or pancreatectomy (n = 29). The PDACs were located in the pancreatic head (n = 19), body (n = 18), and tail (n = 13). The mean maximum diameter of the PDAC on the axial PP images at VMI_70_ was 22.3 ± 11.8 mm (range, 12.5‒43.9 mm). The mean cumulative CTDI_vol_ and DLP of the CECT examinations was 10.8 ± 2.4 mGy and 306.9 ± 80.7 mGy × cm for both the PP and PVP images, respectively.

**Table 1:** Patient demographics, tumor characteristics, and radiation exposure

Parameter	Results
Patient demographics	
Age (years)	68.2 ± 10.4 (44‒88)
Sex	
Men	36 (72.0)
Women	14 (28.0)
Height (cm)	163.7 ± 9.7 (140.3‒180.2)
Body weight (kg)	60.5 ± 10.7 (39.1‒81.3)
Body mass index (kg/m^2^)	22.5 ± 3.2 (16.8‒34.7)
Tumor characteristics	
Location	
Head	19 (38.0)
Body	18 (36.0)
Tail	13 (26.0)
Maximum diameter (mm)	22.3 ± 11.8 (12.5‒43.9)
Radiation exposure	
CTDI_vol_ (mGy)	10.7 ± 2.3 (9.2‒17.6)
DLP (mGy × cm)	306.3 ± 79.1 (210.5‒560.9)

Data are summarized as mean ± standard (range) deviation for continuous variables or as counts (percentages) for categorical variables

*CTDI*_vol_ = volume CT dose index, *DLP* = dose-length product

### Quantitative Image Analysis

**Fig. 2:**
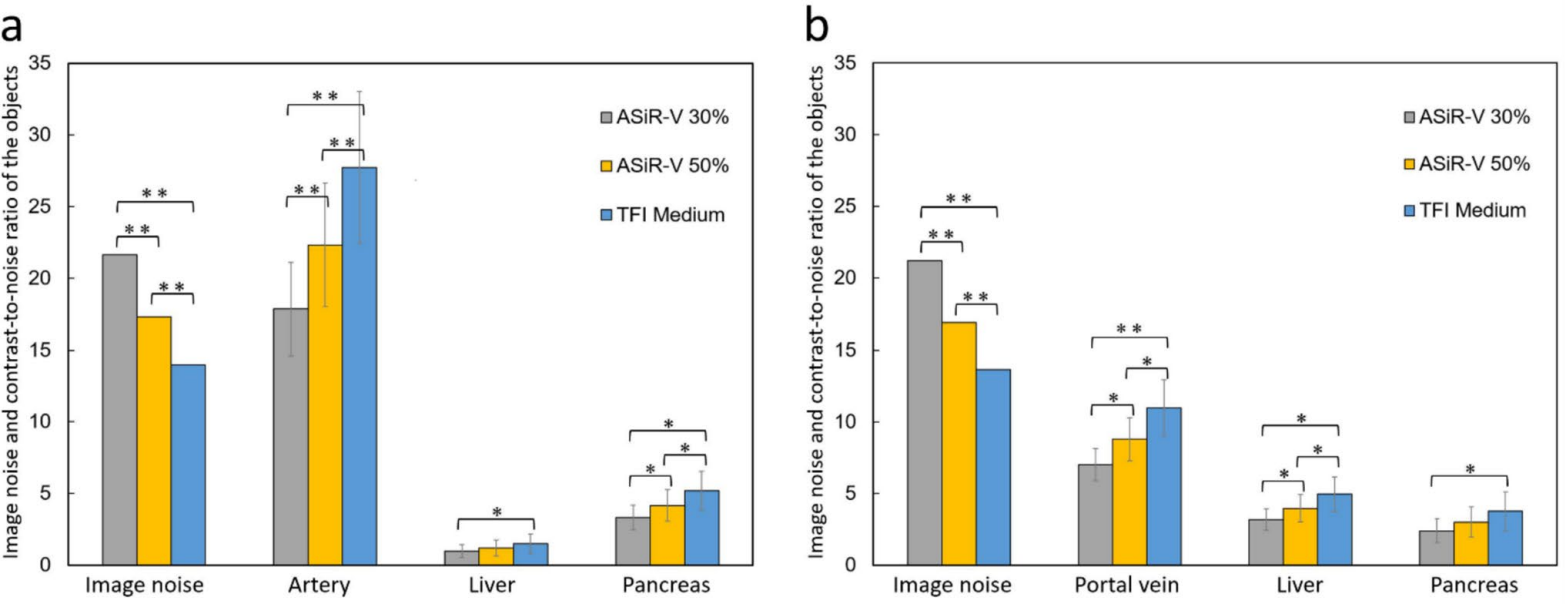
Image noise (standard deviation) of the paraspinal muscle and contrast-to-noise ratio of the aorta, portal vein, liver, and pancreas for each reconstruction algorithm on **(a)** pancreatic phase and **(b)** portal venous phase images of the virtual monochromatic images at 70 kiloelectron-volt. Error bars represent the standard deviations of the means obtained from the 50 patients. ASiR-V, adaptive statistical iterative reconstruction-V; TFI-M, deep learning image reconstruction (TrueFidelity) at medium level. * = significant difference; *P* < 0.05, and ** = significant difference; *P* < 0.01

The CT values of the aorta, portal vein, liver, pancreas, PDAC, and paraspinal muscle were equivalent between the PP and PVP images in VMI_70_ among the three reconstruction algorithms (*P* = 0.82‒0.99) (Table 2). The mean image noise gradually decreased in VMI_70_ with ASiR-V 30%, ASiR-V 50%, and TFI-M, with statistically significant differences in the Bonferroni correction between the PP and PVP images (*P* < 0.001 for all). The CNR of the aorta, portal vein, liver, pancreas, and PDAC were significantly higher between the PP and PVP images in VMI_70_ with TFI-M than in those with ASiR-V 30% and ASiR-V 50% (*P* < 0.001 for all) (Table 3, Fig. 2).

**Table 2:** CT values of the objects on virtual monochromatic images at 70-kiloelectronvolt in each reconstruction algorithms

Parameter	ASiR-V 30%	ASiR-V 50%	TFI-M	*P* Value
Pancreatic phase				
Aorta (HU	439.3 ± 79.3 (305.6‒615.7)	439.4 ± 79.2 (305.7‒614.5)	439.5 ± 79.0 (305.3‒613.1)	0.98
Liver (HU)	76.7 ± 8.5 (65.2‒106.7)	76.7 ± 8.5 (65.1‒106.5)	76.7 ± 8.4 (64.4‒105.8)	0.82
Pancreas (HU)	127.1 ± 18.4 (70.7‒174.9)	127.5 ± 18.3 (70.7‒174.1)	127.1 ± 18.3 (71.5‒173.1)	0.99
PDAC (HU)	60.8 ± 20.1 (17.7‒101.0)	60.9 ± 20.0 (17.4‒100.5)	61.3 ± 20.2 (16.9‒101.3)	0.97
Paraspinal muscle (HU)	56.4 ± 3.6 (48.5‒61.8)	56.4 ± 3.6 (48.4‒61.4)	56.3 ± 3.6 (47.5‒61.1)	0.99
Portal venous phase				
Portal vein (HU)	213.0 ± 23.8 (169.6‒286.9)	212.9 ± 23.7 (169.9‒286.9)	213.5 ± 23.7 (170.5‒287.6)	0.95
Liver (HU)	132.0 ± 12.7 (103.4‒156.9)	132.0 ± 12.6 (104.0‒156.3)	132.1 ± 12.6 (105.1‒156.8)	0.86
Pancreas (HU)	115.6 ± 14.1 (70.3‒151.1)	115.5 ± 14.1 (70.7‒150.2)	115.5 ± 14.1 (70.7‒150.2)	0.99
PDAC (HU)	73.0 ± 28.0 (12.9‒124.0)	73.1 ± 27.9 (13.0‒123.9)	73.5 ± 28.1 (13.8‒124.5)	0.90
Paraspinal muscle (HU)	65.8 ± 5.1 (54.7‒75.6)	65.8 ± 4.8 (55.0‒74.7)	65.7 ± 4.6 (55.5‒73.8)	0.95

Data are presented as mean ± standard deviation. Numbers in parentheses represent the ranges. *ASiR-V*, adaptive statistical iteration reconstruction; *TFI-M*, TrueFidelity image-medium; *PDAC*, pancreatic ductal adenocarcinoma

**Table 3:** Image noise and contrast-to-noise ratio of each object on virtual monochromatic images at 70-kiloelectronvolt in each reconstruction algorithm

Parameter	ASiR-V 30%	ASiR-V 50%	TFI-M	*P* Value
Pancreatic phase				
Image noise	21.6 ± 3.5 (13.8‒29.9)	17.3 ± 3.0 (11.1‒24.3)	13.9 ± 2.4 (8.7‒19.4)	< 0.001
Aorta	17.8 ± 3.3 (12.8‒25.9)	22.3 ± 4.3 (16.1‒32.9)	27.7 ± 5.3 (19.7‒41.7)	< 0.001
Liver	1.0 ± 0.4 (0.2‒2.1)	1.2 ± 0.6 (0.3‒2.6)	1.5 ± 0.7 (0.4‒3.3)	< 0.001
Pancreas	3.3 ± 0.9 (0.6‒4.8)	4.2 ± 1.1 (0.7‒6.0)	5.2 ± 1.4 (0.9‒7.4)	< 0.001
PDAC	3.1 ± 1.1 (1.0‒5.0)	3.9 ± 1.3 (1.2‒6.3)	4.8 ± 1.7 (1.6‒7.3)	< 0.001
Portal venous phase				
Image noise	21.2 ± 2.8 (14.5‒25.6)	16.9 ± 2.3 (11.3‒20.7)	13.6 ± 1.8 (8.9‒17.0)	< 0.001
Portal vein	7.0 ± 1.1 (5.5‒10.5)	8.8 ± 1.5 (6.9‒13.5)	11.0 ± 1.9 (8.8‒17.2)	< 0.001
Liver	3.2 ± 0.7 (2.1‒5.8)	4.0 ± 1.0 (2.6‒7.4)	4.9 ± 1.2 (3.3‒9.5)	< 0.001
Pancreas	2.4 ± 0.8 (0.1‒4.9)	3.0 ± 1.1 (0.1‒6.3)	3.8 ± 1.4 (0.2‒8.1)	< 0.001
PDAC	2.2 ± 1.1 (0.5‒4.6)	2.7 ± 1.4 (0.6‒6.0)	3.4 ± 1.7 (0.8‒7.7)	< 0.001

Measurement data are presented as mean ± standard deviation. Numbers in parentheses represent the ranges

*ASiR-V*, adaptive statistical iteration reconstruction; *TFI-M*, TrueFidelity image-medium; *PDAC*, pancreatic ductal adenocarcinoma

**Fig. 3:**
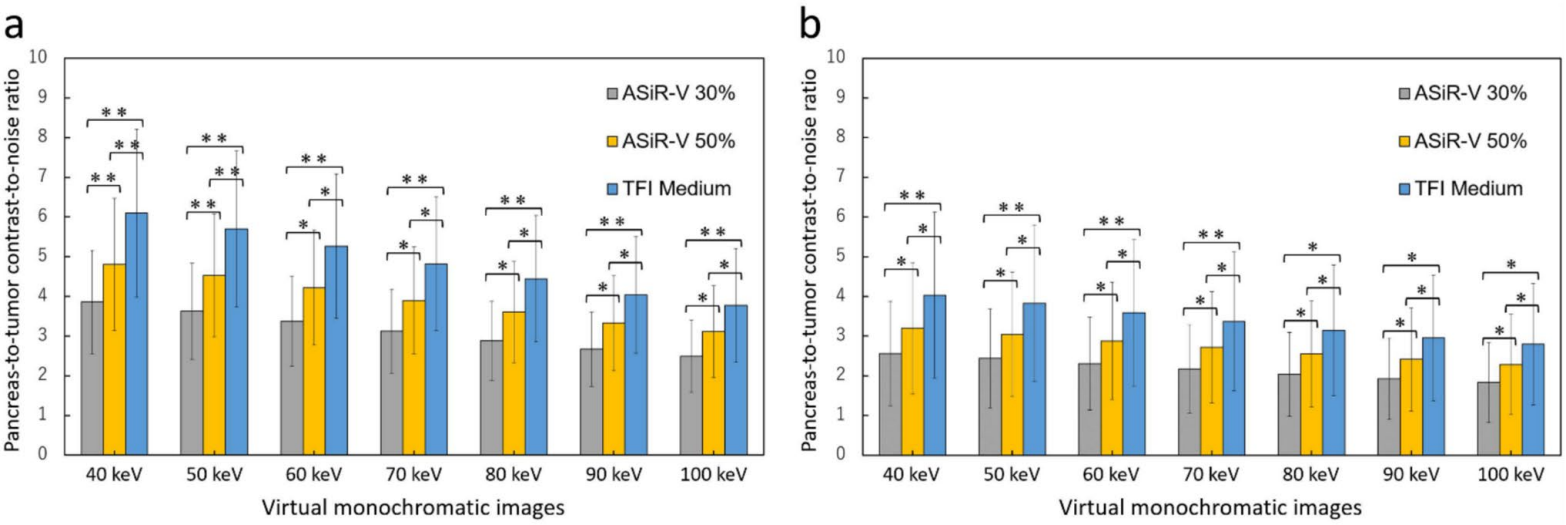
Pancreas-to-tumor contrast-to-noise ratio calculated on **(a)** pancreatic phase and **(b)** portal venous phase images of the virtual monochromatic images at 40–100 kiloelectron-volt. Error bars represent the standard deviations of the means obtained from the 50 patients. ASiR-V, adaptive statistical iterative reconstruction-V; TFI-M, deep learning image reconstruction (TrueFidelity) at medium level. * = significant difference; *P* < 0.05, and ** = significant difference; *P* < 0.01

The pancreas-to-tumor CNR gradually increased with a decrease in monochromatic energy levels in both the PP and PVP images; VMI_40_ provided the highest pancreas-to-tumor CNR (Table 4, Fig. 3). The differences in pancreas-to-tumor CNR between the VMI with TFI-M and those with ASiR-V 30% and ASiR-V 50% were more prominent in the VMI at lower keV.

**Table 4:** Pancreas-to-tumor contrast-to-noise ratio of the pancreatic ductal adenocarcinoma on virtual monochromatic images in each reconstruction algorithm

Parameter	ASiR-V 30%	ASiR-V 50%	TFI-M	*P* Value
Pancreatic phase				
VMI_40_	3.9 ± 1.3 (1.1‒6.1)	4.8 ± 1.7 (1.4‒7.8)	6.1 ± 2.1 (1.9‒9.3)	< 0.001
VMI_50_	3.6 ± 1.2 (1.1‒5.7)	4.5 ± 1.6 (1.3‒7.3)	5.7 ± 2.0 (1.8‒8.7)	< 0.001
VMI_60_	3.4 ± 1.1 (1.0‒5.4)	4.2 ± 1.4 (1.3‒6.8)	5.3 ± 1.8 (1.7‒8.0)	< 0.001
VMI_70_	3.1 ± 1.1 (1.0‒5.0)	3.9 ± 1.3 (1.2‒6.3)	4.8 ± 1.7 (1.6‒7.3)	< 0.001
VMI_80_	2.9 ± 1.0 (0.9‒4.7)	3.6 ± 1.3 (1.2‒5.9)	4.4 ± 1.6 (1.2‒6.9)	< 0.001
VMI_90_	2.7 ± 0.9 (0.8‒4.3)	3.3 ± 1.2 (0.9‒5.4)	4.0 ± 1.5 (0.9‒6.2)	< 0.001
VMI_100_	2.5 ± 0.9 (0.6‒4.1)	3.1 ± 1.2 (0.7‒5.1)	3.8 ± 1.4 (0.7‒6.1)	< 0.001
Portal venous phase				
VMI_40_	2.6 ± 1.3 (0.8‒5.3)	3.2 ± 1.7 (0.9‒6.8)	4.0 ± 2.1 (1.1‒8.7)	< 0.001
VMI_50_	2.4 ± 1.2 (0.7‒5.0)	3.0 ± 1.6 (0.9‒6.4)	3.8 ± 2.0 (1.1‒8.3)	< 0.001
VMI_60_	2.3 ± 1.2 (0.6‒4.8)	2.9 ± 1.5 (0.8‒6.2)	3.6 ± 1.8 (1.0‒8.0)	< 0.001
VMI_70_	2.2 ± 1.1 (0.5‒4.6)	2.7 ± 1.4 (0.6‒5.9)	3.4 ± 1.7 (0.8‒7.7)	< 0.001
VMI_80_	2.0 ± 1.1 (0.4‒4.5)	2.5 ± 1.3 (0.5‒5.7)	3.1 ± 1.6 (0.7‒7.2)	< 0.001
VMI_90_	1.9 ± 1.0 (0.2‒4.3)	2.4 ± 1.3 (0.3‒5.5)	3.0 ± 1.6 (0.4‒6.9)	< 0.001
VMI_100_	1.8 ± 1.0 (0.1‒4.2)	2.3 ± 1.3 (0.1‒5.3)	2.8 ± 1.5 (0.2‒6.5)	< 0.001

Measurement data are presented as mean ± standard deviation. Numbers in parentheses represent the ranges

*ASiR-V* = adaptive statistical iteration reconstruction, Veo; *TFI-M* = True Fidelity image-medium; *VMI* = virtual monochromatic image

The measurements of maximum diameter of the PDAC on PP images are presented in Table 5. The measurements of the maximum diameter of the PDAC were similar between on VMI_40‒100_ with three reconstruction algorithms (22.0‒23.0 mm for ASiR-V 30%; 22.0‒22.7 mm for ASiR-V 50%; 21.8‒22.5 mm for TFI-M) and were shorter as the energy levels decreased. TFI-M tended to have a smaller variation in measurements compared with ASiR-V 30%, even when the monochromatic energy levels changed. Inter-reader agreement for the measurements of maximum diameter of the PDAC were good (ICC, 0.78‒0.84 for ASiR-V 30%; 0.81‒0.85 for ASiR-V 50%; 0.80‒0.87 for TFI-M).

**Table 5:** Maximum diameter of pancreatic ductal adenocarcinoma in virtual monochromatic images on pancreatic phase images in each reconstruction algorithm

Parameter	ASiR-V 30%	ASiR-V 50%	TFI-M
Maximum diameter (mm)			
VMI_40_	22.2 ± 13.2 (11.6‒45.8)	22.0 ± 11.9 (11.9‒43.6)	21.8 ± 11.7 (12.1‒43.7)
ICC	0.83	0.85	0.87
VMI_50_	22.0 ± 12.8 (11.2‒43.9)	22.4 ± 12.0 (12.0‒43.9)	22.0 ± 11.9 (11.8‒43.8)
ICC	0.84	0.85	0.83
VMI_60_	22.4 ± 12.5 (12.1‒44.1)	22.3 ± 11.7 (12.1‒43.8)	22.1 ± 11.8 (12.5‒43.8)
ICC	0.81	0.84	0.85
VMI_70_	22.5 ± 12.2 (12.2‒44.3)	22.4 ± 11.9 (12.5‒44.1)	22.3 ± 11.8 (12.5‒43.9)
ICC	0.83	0.81	0.82
VMI_80_	22.6 ± 12.2 (12.0‒44.2)	22.5 ± 11.9 (12.2‒44.1)	22.3 ± 11.9 (12.7‒44.3)
ICC	0.84	0.81	0.80
VMI_90_	23.0 ± 12.4 (11.5‒45.1)	22.5 ± 11.8 (12.3‒44.6)	22.5 ± 12.0 (12.3‒44.2)
ICC	0.78	0.83	0.84
VMI_100_	22.9 ± 12.4 (11.7‒45.0)	22.7 ± 12.2 (11.9‒44.4)	22.5 ± 12.0 (12.1‒6.1)
ICC	0.81	0.82	0.82

Measurement data are presented as mean ± standard deviation. Numbers in parentheses represent the ranges

*ASiR-V* = adaptive statistical iteration reconstruction, Veo; *TFI-M* = True Fidelity image-medium; *VMI* = virtual monochromatic image; *ICC*, intraclass correlation coefficient

### Qualitative Image Analysis

**Fig. 4:**
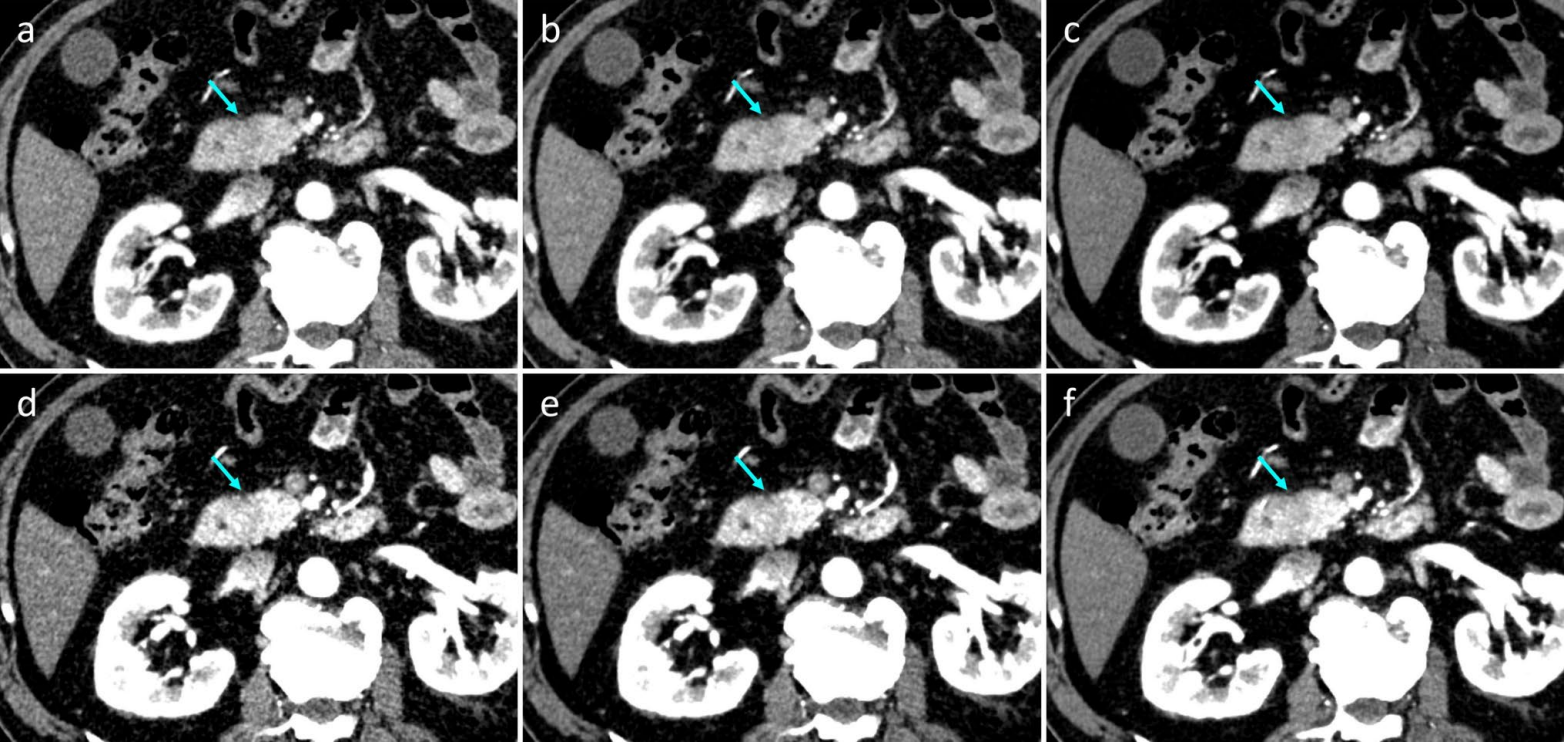
Virtual monochromatic images (VMIs) on pancreatic phase in a 52-year-old male patient with pancreatic ductal adenocarcinoma of the pancreatic head reconstructed with **(a, d)** ASiR-V 30%, **(b, e)** ASiR-V 50%, and **(c, f)** TFI-M at 70 keV (upper row) and 40 keV (lower row). The CNR and conspicuity of the PDAC are superior on VMI at 40 keV compared with that at 70 keV. Image noises are superior on VMIs with TFI-M to ASiR-V both at 70 keV and 40 keV. *ASiR-V*, adaptive statistical iterative reconstruction; *TFI-M*, deep learning image reconstruction (TrueFidelity) at the medium level. The conspicuities of PDAC were; **(a)** 3 and 2, **(b)** 3 and 3, **(c)** 3 and 3, **(d)** 2 and 2, **(e)** 3 and 3, **(f)** 4 and 4 for the two readers

**Fig. 5:**
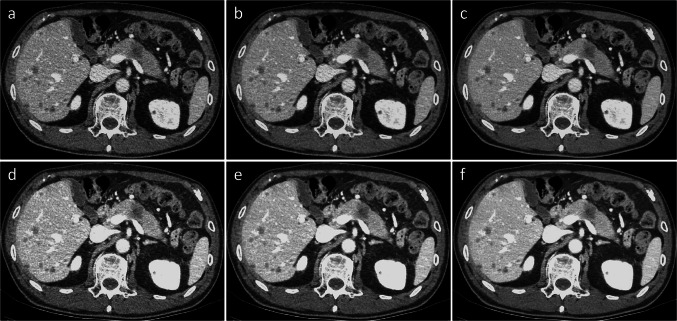
Virtual monochromatic images (VMIs) on portal venous phase in a 73-year-old male patient with pancreatic ductal adenocarcinoma of the pancreatic body and multiple liver metastases reconstructed with **(a, d)** ASiR-V 30%, **(b, e)** ASiR-V 50%, and **(c, f)** TFI-M at 70 keV (upper row) and 40 keV (lower row). The CNR and conspicuity of the PDAC and liver metastases are superior on VMI at 40 keV compared with that at 70 keV. Image noises are superior on VMIs with TFI-M to ASiR-V both at 70 keV and 40 keV. *ASiR-V*, adaptive statistical iterative reconstruction; *TFI-M*, deep learning image reconstruction (TrueFidelity) at the medium level. The conspicuities of PDAC were; **(a)** 4 and 5, **(b)** 4 and 5, **(c)** 4 and 5, **(d)** 4 and 5, **(e)** 5 and 5, **(f)** 5 and 5 for the two readers

Qualitative analysis was performed using VMI_70_ and VMI_40_ with all four reconstruction algorithms on the PP and PVP according to the results of the quantitative analysis (Table 6). Regarding image noise, soft-tissue sharpness, and overall image quality, TFI-M demonstrated significantly better image quality than ASiR-V 30% (*P* < 0.001 for all) and ASiR-V 50% (*P* < 0.05 for all) in both VMI_70_ and VMI_40_ on PP and PVP images (Figs. 4 and 5). On vessel conspicuity, TFI-M and ASiR-V 50% had significantly higher scores than ASiR-V 30% in both VMI_70_ and VMI_40_ on PP and PVP images (*P* < 0.001 for all). On conspicuity of PDAC, TFI-M and ASiR-V 50% had significantly higher scores than ASiR-V 30% in both VMI_70_ and VMI_40_ on PVP images (*P* < 0.001 for all). Compared with VMI_70_, VMI_40_ had inferior image quality; however, VMI_40_ with TFI-M showed relatively superior vessel conspicuity and conspicuity of PDAC on both the PP and PVP images. The ĸ values of the qualitative analysis ranged from 0.61 to 0.78, indicating substantial agreement between the two readers.

**Table 6:** Qualitative image analysis on virtual monochromatic images in each reconstruction algorithm

Parameter	ASiR-V 30%	ASiR-V 50%	TFI-M	*P* Value
Pancreatic phase				
VMI_70_				
Image noise	3.4 ± 0.7 (0.69)	3.9 ± 0.8 (0.70)	4.3 ± 0.6 (0.72)	< 0.001
Soft tissue sharpness	3.5 ± 0.5 (0.72)	3.9 ± 0.6 (0.71)	4.2 ± 0.7 (0.69)	< 0.001
Vessel conspicuity *	3.3 ± 0.6 (0.78)	3.8 ± 0.6 (0.71)	3.8 ± 0.5 (0.76)	< 0.01
Conspicuity of PDAC	3.6 ± 0.8 (0.72)	3.9 ± 0.7 (0.71)	4.2 ± 0.8 (0.74)	< 0.001
Overall image quality	3.2 ± 0.8 (0.77)	3.5 ± 0.8 (0.75)	3.8 ± 0.6 (0.78)	< 0.001
VMI_40_				
Image noise	2.8 ± 0.9 (0.64)	3.4 ± 0.8 (0.69)	4.0 ± 0.8 (0.73)	< 0.001
Soft tissue sharpness	3.6 ± 0.6 (0.70)	4.0 ± 0.7 (0.73)	4.3 ± 0.6 (0.72)	< 0.001
Vessel conspicuity *	3.7 ± 0.7 (0.71)	4.1 ± 0.6 (0.73)	4.2 ± 0.6 (0.71)	< 0.01
Conspicuity of PDAC	4.0 ± 0.7 (0.69)	4.3 ± 0.7 (0.72)	4.5 ± 0.8 (0.73)	< 0.001
Overall image quality	2.9 ± 1.0 (0.68)	3.3 ± 0.8 (0.69)	3.6 ± 0.7 (0.68)	< 0.001
Portal venous phase				
VMI_70_				
Image noise	3.5 ± 0.8 (0.67)	3.9 ± 0.7 (0.71)	4.4 ± 0.7 (0.70)	< 0.001
Soft tissue sharpness	3.7 ± 0.6 (0.67)	3.9 ± 0.7 (0.68)	4.2 ± 0.8 (0.64)	< 0.001
Vessel conspicuity*	3.2 ± 0.7 (0.74)	3.7 ± 0.7 (0.74)	3.6 ± 0.7 (0.71)	< 0.001
Conspicuity of PDAC*	3.3 ± 0.7 (0.75)	3.7 ± 0.8 (0.72)	3.6 ± 0.6 (0.75)	< 0.01
Overall image quality	3.5 ± 0.7 (0.74)	3.7 ± 0.6 (0.71)	4.1 ± 0.6 (0.70)	< 0.001
VMI_40_				
Image noise	2.9 ± 0.7 (0.61)	3.3 ± 0.7 (0.68)	3.6 ± 0.8 (0.64)	< 0.001
Soft tissue sharpness	3.6 ± 0.7 (0.74)	3.9 ± 0.7 (0.77)	4.1 ± 0.7 (0.71)	< 0.001
Vessel conspicuity *	3.7 ± 0.8 (0.61)	4.2 ± 0.7 (0.64)	4.2 ± 0.7 (0.69)	< 0.001
Conspicuity of PDAC *	3.6 ± 0.7 (0.75)	3.9 ± 0.6 (0.70)	3.9 ± 0.5 (0.71)	< 0.01
Overall image quality	3.1 ± 0.9 (0.67)	3.4 ± 0.7 (0.66)	3.8 ± 09 (0.62)	< 0.001

Measurement data are presented as mean ± standard deviation. Numbers in parenthesis are κ values between the two readers

*ASiR-V* = adaptive statistical iteration reconstruction, Veo; *TFI-M* = True Fidelity image-medium; *VMI* = virtual monochromatic image; *PDAC* = pancreatic ductal adenocarcinoma

^*^Values did not significantly differ (*P* > 0.05) between ASiR-V 50% and TFI-M

Twenty-nine of 50 patients underwent surgical resection in our study, and 16 of 29 patients received neoadjuvant chemotherapy. Among the 29 patients who underwent curative intent resection, R0, R1, and R2 resection were achieved in 23 (79.3%), 5 (17.2%), and one (3.5%) patients, respectively. The mean sensitivity, specificity, and AUC values of VMI_40_ and VMI_70_ with TFI-M for the diagnosis of R0 resection was 89.1% and 84.8%, 83.3% and 75.0%, and 0.93 and 0.90, respectively with no significant differences. Interobserber agreements were substantial for VMI_40_ (0.63) and VMI_70_ (0.61) with TFI-M.

### Phantom Experiment

**Fig. 6:**
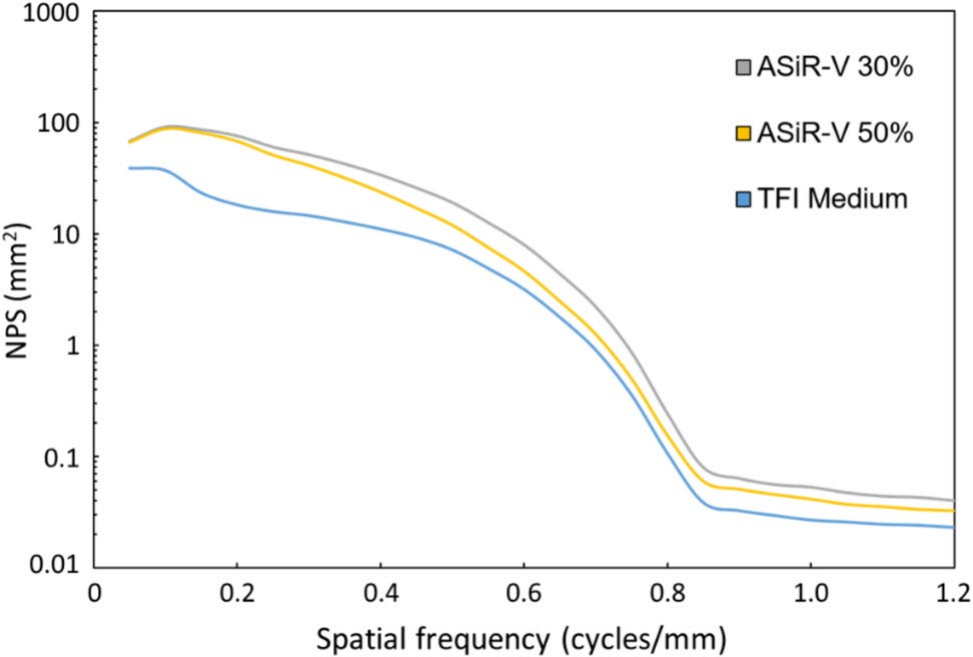
Graph shows NPS curves for the three CT protocols. Compared with the two iterative reconstruction algorithms (ASiR-V 30% and ASiR-V 50%), TFI-M algorithm yielded relatively more pronounced noise reduction at lower spatial frequencies (ie, broad texture features) than at higher spatial frequencies (ie, fine detailed texture features)

NPS curves for the VMI_70_ reconstructed with FBP, ASiR-V 30%, ASiR-V 50%, and TFI-M algorithms are shown in Fig. 6. VMI_70_ with TFI-M yielded quantifiable noise reduction compared with VMI_70_ with FBP, ASiR-V 30%, and ASiR-V 50% across the entire spectrum of spatial frequencies at both radiation dose levels. This effect was more prominent at higher frequencies than at higher frequencies. No spatial frequency shift was observed in the TFI-M reconstruction algorithm.

## Discussion

This study evaluated the effectiveness of VMI reconstruction using commercially available DLIR in DECT for PDAC detection and assessment. Our findings indicate that DLIR (TFI-M) significantly improves image quality and reduces image noise in VMIs compared to conventional iterative reconstruction algorithms (ASiR-V 30% and ASiR-V 50%), especially at lower keV levels. These results suggest that VMIs with DLIR can improve the diagnostic performance of PDAC imaging.

A major issue in diagnosing PDAC is the insufficient contrast between the tumor and pancreatic parenchyma on conventional CECT imaging, which may hinder the accurate detection of PDAC [5, 6]. Previous studies have shown that low-keV VMIs from DECT enhance pancreas-to-tumor contrast by increasing iodine attenuation and improving tumor visibility [11–13]. However, increased image noise associated with lower keV levels may compromise diagnostic utility [14, 15]. In this study, we found that TFI-M significantly reduced image noise while preserving image texture, particularly at 40 keV, where the pancreas-to-tumor CNR was the highest. This suggests that TFI-M can mitigate noise-related issues in lower-keV VMI and improve tumor detection.

Our quantitative analysis showed that TFI-M consistently reduced image noise in both PP and PVP images compared to ASiR-V, with significant differences across all keV levels. These results are consistent with previous reports demonstrating DLIR's ability to reduce noise while preserving image details in other anatomical regions [17–19]. Importantly, our findings suggest that TFI-M may improve low-keV VMI by providing a clearer distinction between PDAC and surrounding pancreatic parenchyma, potentially enhancing tumor detection and characterization [14, 15]. The improved CNR with TFI-M at lower keV levels, particularly at 40 keV, is clinically relevant for PDAC diagnosis and management. Higher contrast between pancreatic parenchyma and PDAC at these energy levels may enable earlier detection of smaller or less conspicuous tumors compared to conventional CT. The phantom experiment further illustrated the noise reduction capabilities of the TFI-M. NPS analysis showed that TFI-M effectively reduced noise across all spatial frequencies, especially at lower frequencies, where abdominal diagnostic information is most critical. These results demonstrate the potential of TFI-M to improve image quality without introducing artifacts or frequency shifts that could affect diagnostic accuracy.

Our qualitative analysis confirmed the superiority of TFI-M over ASiR-V 30% and ASiR-V 50% in subjective image quality, with significant improvements in image noise, soft tissue sharpness, and PDAC conspicuity. While VMI at 40 keV exhibited increased noise and slightly lower overall image quality than VMI at 70 keV, regardless of the reconstruction algorithm, TFI-M maintained diagnostic acceptability compared to ASiR-V 30% and ASiR-V 50%. Additionally, vessel conspicuity was better in VMIs with TFI-M than in those with ASiR-V 30% and ASiR-V 50%. Previous reports showed that DLIR improves the visualization of small vessels compared to iterative reconstruction algorithms because it reduces image noise while maintaining image contrast, resolution, and texture [24, 25]. Substantial interobserver agreement supports the reliability of these qualitative findings.

These findings have important clinical implications. The improved CNR with TFI-M at lower keV levels, particularly at 40 keV, is highly relevant for PDAC diagnosis and therapeutic management. This improvement is crucial for PDAC because early detection and accurate staging are key to improving surgical outcomes and patient survival rates (3, 4). Accurate measurement of tumor size is also important for assessing the treatment response after chemotherapy. Concordance in treatment response assessments between radiologists can improve outcomes when providing the most accurate and appropriate medical care. Additionally, a significant reduction in image noise with TFI-M could allow the acquisition of high-quality images at lower radiation doses, addressing concerns regarding cumulative radiation exposure in patients undergoing repeated imaging for cancer staging and follow-up [10]. Although our results showed no significant difference in diagnostic performance between VMI_40_ and VMI_70_ with TFI-M in our study, further research including a larger number of patients is required to clarify the potential benefits of DLIR in the evaluation of surgical resectability of PDAC.

Despite these promising results, our study had some limitations. First, the study population was relatively small, and large-scale studies are necessary to validate our findings. Additionally, the retrospective design may have introduced selection bias, although our inclusion criteria aimed to minimize this risk. Second, tumor staging and surgical resectability were not evaluated. Although we demonstrated that DLIR improves the image quality in pancreatic DECT, further research is needed to assess its impact on clinical outcomes, including the accuracy of PDAC diagnosis and staging. Third, we did not include dice similarity coefficient analysis and edge rise distance analysis to provide valuable insights for the quantitative evaluation of tumor boundary delineation, which may require further research. Fourth, we evaluated a single DLIR algorithm (TrueFidelity), which is one of the commercially available algorithms. Since DLIR techniques significantly differ between vendors in terms of training data, network architecture, and implementation, direct comparison between different DLIR algorithms was not performed in this study. Finally, this study employed only a fast kilovoltage-switching DECT scanner from a single vendor; it remains uncertain whether the results of our study can be replicated using other DECT scanners. Future studies using other DECT platforms that employ different material decomposition algorithms with different datasets are required.

In conclusion, DLIR significantly improved the image quality of VMIs in DECT by reducing image noise and improving CNR, particularly at lower keV levels. The improved CNR with TFI-M at lower keV levels, particularly at 40 keV, is highly relevant for PDAC diagnosis at earlier stage and more accurate tumor staging. Future studies should focus on validating these findings and exploring the clinical benefits of incorporating DLIR into routine pancreatic imaging.

## Data Availability

The datasets used and/or analyzed during the current study are available from the corresponding author on reasonable request.
